# The Effect of Dietary Pattern on Metabolic Syndrome in a Suburban Population in Shanghai, China

**DOI:** 10.3390/nu15092185

**Published:** 2023-05-04

**Authors:** Lanxin Wei, Jing Fan, Ruihua Dong, Mei Zhang, Yonggen Jiang, Qi Zhao, Genming Zhao, Bo Chen, Jing Li, Shaojie Liu

**Affiliations:** 1 Key Laboratory of Public Health Safety of Ministry of Education, School of Public Health, Fudan University, Shanghai 200032, China; lxwei21@m.fudan.edu.cn (L.W.); 21111020063@m.fudan.edu.cn (J.F.); ruihua_dong@fudan.edu.cn (R.D.); zhaoqi@shmu.edu.cn (Q.Z.); gmzhao@shmu.edu.cn (G.Z.); chenb@fudan.edu.cn (B.C.); 2 Zhongshan Community Health Care Center, Songjiang District, Shanghai 201613, China; 18918287235@163.com; 3 Songjiang District Center for Disease Control and Prevention, Shanghai 201620, China; sjjktj@hotmail.com

**Keywords:** metabolic syndrome, posteriori dietary pattern, priori dietary pattern, cross-section study

## Abstract

Metabolic syndrome (MetS) is recognized as one of the most severe non-communicable chronic diseases. Diet plays an essential role in the development and exacerbation of MetS. Thus, this study aimed to investigate the relationship between dietary patterns and MetS in a suburban population in Shanghai, China. Data were collected on the Zhongshan community from the Shanghai Suburban Adult Cohort and Biobank (SSACB) study between May and September 2017. A total of 5426 participants who completed the questionnaire investigation, physical measurements, and biological sample collection were effectively enrolled in this study. Both posteriori and priori methods were utilized to generate different dietary patterns, including the dietary approaches to stop hypertension (DASH) and Mediterranean diet (MD). The prevalence of MetS in this study was 22.47%. Compared to the reference, dietary patterns with a higher intake of “dairy and fruits” and “coarse cereals and soy products” had protective effects on MetS (*p* < 0.05). However, no significant correlation with MetS was observed for DASH and MD. Our study recommends higher consumption of fruits, coarse cereals, and soy products, which was associated with a lower prevalence of MetS in the suburban population of Shanghai. The correlation of DASH and MD with MetS in the Chinese population requires further exploration.

## 1. Introduction

Metabolic syndrome (MetS) is a collection of intricate syndromes typified by metabolic disorders, including being overweight, obesity, hypertension, hyperglycemia, and dyslipidemia, as defined by the diagnostic criteria of the Chinese Diabetes Society (CDS). The prevalence of MetS has increased significantly in several countries. In Korea, the prevalence of MetS increased from 24.9% in 1998 to 37% in 2012 [1]. In China, the prevalence of MetS increased from 13.7% in 2000 to 24.2% in 2012 [2,3]. In the United States, approximately one-third of adults have MetS [4], whereas in Russia, the prevalence was 23.1% and 11.0% among women and men, respectively [5]. MetS has been regarded as one of the most serious non-communicable chronic diseases and is a risk factor for type 2 diabetes and cardiovascular disease (CVD) [6,7]. Several studies have found that the prevalence of diabetes and CVD in the population with MetS was five-fold and doubled-fold higher than that without MetS, respectively [8,9]. The rapid increase in MetS prevalence may be attributed to changes in population behavior patterns in modern society [10,11]. Among these patterns, diet plays an essential and independent role in the incidence and development of MetS [12,13]. Hence, it is important and meaningful to explore the association between diet consumption and the prevalence of MetS.

Several studies have assessed the effects of single foods or food compositions on MetS [14,15]. However, given the synergistic effects of different foods, dietary patterns may be more appropriate to evaluate the association between food intake and MetS. Two methods are commonly used for evaluating dietary patterns: the a priori and a posteriori methods. A priori dietary patterns, also known as dietary indices, can intuitively provide a comprehensive explanation of complex results. Various dietary indices have been developed to investigate the relationship between MetS and dietary patterns, including the dietary approaches to stop hypertension (DASH) diet, the Mediterranean diet, and the Healthy Eating Index [16,17,18,19]. Conversely, the a posteriori method analyses dietary data using cluster analysis, factor analysis, or latent class analysis to identify and generalize different dietary patterns. This method is more flexible than the a priori method, with fixed food items and is closer to reality. Thus, both methods have individual advantages in exploring the association between diet and disease. However, most studies have only used one method to investigate the relationship between dietary patterns and MetS [20,21]. Therefore, it is necessary to adopt both methods to comprehensively assess the relationship between dietary patterns and MetS.

In this study, we employ both a priori and a posteriori methods to examine the association between dietary patterns and MetS in a suburban population in China.

## 2. Subjects and Methods

### 2.1. Study Population

**Figure 1:**
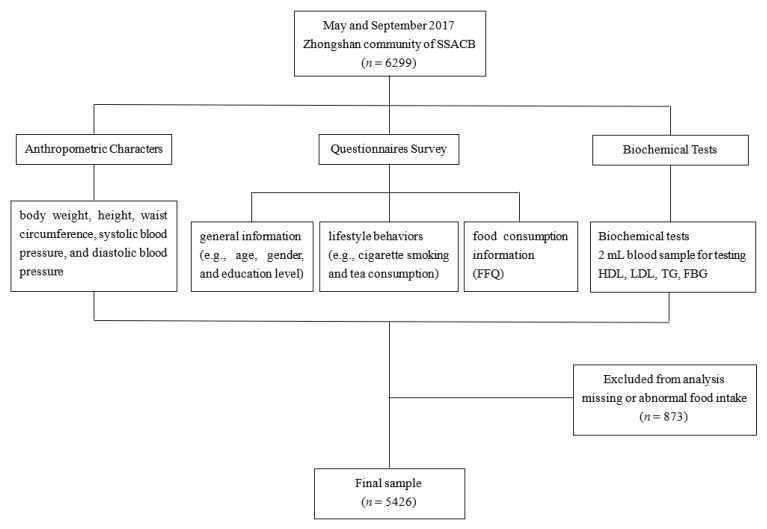
The flowchart of this study.

The study subjects were recruited between May and September 2017 from the Zhongshan community from the Shanghai Suburban Adult Cohort and Biobank (SSACB) [22]. The overarching objective of this cohort is to identify the environmental, lifestyle, and genetic risk factors for non-communicable chronic diseases in adults residing in the suburbs of Shanghai. The cohort details have been described previously [22]. Briefly, a stratified clustered sampling design was employed to obtain data from participants aged between 20 to 74 years old, who were randomly selected through a multistage sampling method. We selected study participants from the Zhongshan community from the SSACB. Participants were excluded if: (1) they lacked a food frequency questionnaire (FFQ) or consumption data on cooking oil and condiments; (2) their self-reported energy intake was less than 800 kcal/d or more than 4000 kcal/d for males and less than 500 kcal/d or more than 3500 kcal/d for females; (3) they had abnormal intake of a single food (exceeding 1000 g/d). Ultimately, a total of 5426 participants were deemed eligible for our study. The flowchart of this study was showed in Figure 1. The Medical Research Ethics Committee of the School of Public Health, Fudan University, reviewed and approved the study (No.: IRB#2016-04-0586), and all the research subjects provided informed consent before participating in the investigation.

### 2.2. Questionnaire Survey

Trained investigators conducted face-to-face interviews to collect data, via questionnaire surveys, from all participants. The surveys captured general information (e.g., age, gender, education level, marital status, and retirement status), lifestyle behaviors (e.g., cigarette smoking, alcohol and tea consumption, and physical activity), as well as food consumption information via a 29 food group-based FFQ, which included rice and grain products, among other food types. The reliability and validity of the FFQ was previously established [23]. Briefly, the FFQ’s reliability was assessed for 152 participants selected randomly from the Zhongshan community in the SSACB by comparing two FFQ surveys taken 12 months apart. Concurrently, the FFQ’s validity was evaluated for 165 participants by comparing the FFQ data with 3-day 24-hour dietary recalls. The results demonstrated a strong correlation coefficient for both food groups (reliability: 0.36–0.54; validity: 0.20–0.41) and nutrients (reliability: 0.39–0.60; validity: 0.12–0.42).

### 2.3. Anthropometric Characters

Trained technologists measured the anthropometric characteristics, including body weight, height, waist circumference, systolic blood pressure, and diastolic blood pressure, following the national standard WS/T424-2013. Specifically, height was measured using a column height meter with an accuracy of 0.1 cm, while weight was measured using an electronic weight meter with an accuracy of 0.1 kg. Waist circumference was measured using a flexible tape at the midpoint between the iliac crest and the last rib, with the subject at minimal respiration, and had an accuracy of 0.1 cm. Systolic and diastolic blood pressure were measured at the right arm of seated study participants after a five minute rest period, with readings recorded to the nearest 2 mmHg. To ensure accuracy, all physical measurements were performed three times, and the mean values were used for analysis.

### 2.4. Biochemical Tests

Trained technologists collected a 2 mL blood sample from all the study participants and drew it into tubes containing EDTA. The samples were then transported to the Shanghai Dian Diagnosis Innovation Professional clinical laboratories for testing. Biochemical tests were conducted to measure the cardiovascular-related indicators for determining the prevalence of MetS in individuals. Specifically, we measured high-density lipoprotein (HDL), low-density lipoprotein (LDL), triglyceride (TG), and fasting blood glucose (FBG).

### 2.5. Covariate Assessment

We calculated the total hours of metabolism equivalent (MET) per week, based on the Compendium of Physical Activity [24]. Energy intake per day was estimated using the FFQ. Body mass index (BMI, kg/m^2^) was determined using the formula = weight (kg) / height^2^ (m^2^), and then classified into four categories based on the Working Group on Obesity in China: underweight (< 18.5 kg/m^2^), normal (18.5–23.9 kg/m^2^), overweight (24.0–27.9 kg/m^2^), and obesity (≥ 28.0 kg/m^2^). Education level was categorized as primary school and below, junior high school, high school and above. Marital status was classified as married or other. To identify the presence of MetS, we used the criteria set by the Chinese Diabetes Society (CDS), which defines MetS as having three or more of the following five criteria: (1) elevated waist circumference: waist circumference ≥ 90 cm in males, ≥ 85 cm in females; (2) hyperglycemia status: fasting blood glucose (FBG) ≥ 6.1 mmol/L or a diabetes diagnosis; (3) hypertension status: systolic/diastolic blood pressure ≥ 130/85 mmHg or a hypertension diagnosis; (4) elevated triglyceridemic: triglyceridemic (TG) ≥ 1.7 mmol/L; (5) reduced HDL: HDL < 1.04 mmol/L [25].

### 2.6. Analysis of Dietary Patterns

#### 2.6.1. A posteriori Dietary Pattern Analysis

Both factor analysis and cluster analysis were utilized to identify the dietary patterns of the study participants. The procedures are explained in detail below.

First, the average daily intake for each food group was subjected to a cluster analysis using the K-mean method to identify dietary patterns among the study participants. The analysis was performed with 50 iterations, centering results on zero, and the subjects were divided into four groups based on the Euclidean distance between the observations.

Second, a factor analysis was conducted to identify dietary patterns. The adequacy of the sample was first verified using the Kaiser–Meyer–Olkin (KMO) index and Bartlett’s test of sphericity. Principal component analysis was then employed to extract factors, followed by orthogonal rotation. The factors with eigenvalues greater than one were retained. Only absolute values of factor loading > 0.2 were included in the analysis, as high factor loads indicated a strong relationship with the identified factors. Factor scores were further categorized into four quartiles, where high scores indicated a greater adherence to the identified dietary pattern.

#### 2.6.2. A priori Dietary Pattern Analysis

The adherence to two diet quality scores for the dietary approaches to stop hypertension (DASH) diet and the Mediterranean diet (MD) were calculated. The DASH and MD scores ranged from 0 to 9, with higher scores indicating greater adherence to the respective dietary patterns.

The DASH diet score for each participant was determined using the formula developed by Mellen et al. [26]. The score comprised of nine nutrients (total fat, saturated fat, protein, fiber, cholesterol, calcium, magnesium, sodium, and potassium), with micronutrient goals reported per 1000 kcal. Each nutrient was assigned a score of 0, 0.5, or 1 based on whether the target was not met, partially met, or fully met, respectively. The DASH score was then calculated as the sum of all the nutrient targets met by the individual (Supplementary Table S1).

The traditional MD score was originally developed by Trichopoulou et al. [27], to assess adherence to the Mediterranean diet in a Greek population. Fung et al. [28] later revised this score and developed the alternate Mediterranean diet score, which included vegetables, fruits, nuts, whole grains, legumes, fish, the monounsaturated/saturated fat ratio, red and processed meats, and alcohol. In our study, the MD score for each participant was calculated using the formula developed by Fung et al. The total MD score was computed as the sum of the nutrient targets met, with a score of 1 assigned to the consumption of food groups considered beneficial to health at or above the sex-specific median (vegetables, fruits, nuts, whole grains, legumes, fish, and the monounsaturated/saturated fat ratio), below the median for food groups presumed to be detrimental to health (red and processed meats), and moderate ethanol consumption (5–15 g/d in females and 15–25 g/d in males) (Supplementary Table S2).

### 2.7. Statistical Analysis

Continuous variables were reported as mean ± SD and categorical variables were presented as frequency (%). Balance tests for continuous and categorical variables were conducted using the Student *t*-test and chi-square, respectively. Non-conditional logistic regression analysis was performed to examine the relationship between each diet pattern score and MetS. All statistical analyses were conducted using IBM SPSS Statistics software Version 20.0. Two-sided *p* values < 0.05 were considered statistically significant.

## 3. Results

### 3.1. Personal Characteristics and Prevalence of MetS

Table 1 displays the personal characteristics of the study participants stratified by MetS status. Out of the total sample of 5426 participants, 1219 were diagnosed with MetS, while 4207 were classified as non-MetS. The prevalence of MetS was 22.47%. Statistically significant differences (*p* < 0.05) were observed between the two groups in terms of age, BMI, physical activity, gender, retirement status, education level, cigarette smoking, alcohol drinking, and tea consumption. Furthermore, individuals with MetS exhibited higher levels for waist circumference, fasting blood glucose, systolic blood pressure, diastolic blood pressure, triglycerides, HDL-C, and LDL-C compared to those without MetS (*p* < 0.05).

### 3.2. A posteriori Dietary Pattern Analysis

We classified 29 food items from the FFQ into 19 predefined food groups for the purpose of cluster analysis (Supplementary Table S3). As indicated in Table 2, the resulting four clusters were labeled as follows: “snacks and beverages” dietary pattern (cluster I with *n* = 291), “grains and vegetables” dietary pattern (cluster II with *n* = 1051), “balanced” dietary pattern (cluster III with *n* = 2722), and “dairy and fruits” dietary pattern (cluster IV with *n* = 1362).

**Figure 2:**
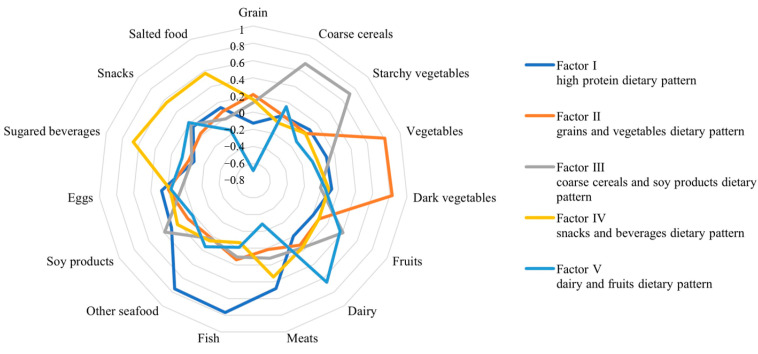
Radar chart for the different dietary patterns from the factor analysis.

Both the Kaiser–Meyer–Olkin index (0.711) and Bartlett’s test (*p* < 0.001) confirmed the suitability of the data for factor analysis. We identified five distinct dietary patterns among the study participants and labeled them as follows: “high protein” dietary pattern (factor I), “grains and vegetables” dietary pattern (factor II), “coarse cereals and soy products” dietary pattern (factor III), “snacks and beverages” dietary pattern (factor IV), and “dairy and fruits” dietary pattern (factor V), based on the highest factor loading for food items and interpretability (Figure 2 and Supplementary Table S4). The variance explained by factor I, factor II, factor III, factor IV, and factor V was 10.933%, 9.869%, 9.556%, 9.062%, and 8.533%, respectively.

### 3.3. A priori Dietary Pattern Analysis

As presented in Table 3, the present results revealed that study participants had a priori dietary pattern score of 1.48 (SD: 1.14) for DASH and 3.93 (SD: 1.59) for MD. Notably, no statistically significant differences in the DASH and MD scores were observed between the study populations with and without MetS (*p* > 0.05).

### 3.4. Association between Dietary Patterns and MetS

Table 4 presents the results of the non-conditional logistic regression analysis examining the association between the a posteriori dietary patterns and MetS. The results of the cluster analysis revealed that the study participants with the “dairy and fruits” dietary pattern (cluster IV) had a protective effect against MetS compared to those with the “balanced” dietary pattern (cluster I) in both unadjusted and adjusted models, with OR values of 0.66 (95% CI: 0.56, 0.78) and 0.81 (95% CI: 0.66, 0.98), respectively. For factor analysis, in the unadjusted model, a higher score for the “coarse cereals and soy products” and “dairy and fruits” dietary pattern was associated with a significantly lower risk of MetS, with OR values of 0.67 (95% CI: 0.56, 0.80; *p*_trend_ < 0.001) and 0.60 (95% CI: 0.50, 0.72; *p*_trend_ < 0.001), respectively. After adjusting for covariates, a higher score for the “coarse cereals and soy products” dietary pattern was still associated with a significantly lower risk of MetS, with an OR value of 0.74 (95%CI: 0.61, 0.91; *p*_trend_ = 0.007).

The non-conditional logistic regression analysis examining the relationship between the a priori dietary patterns and MetS is presented in Table 5. Our findings revealed no significant associations of the DASH and MD scores with MetS (*p* > 0.05).

## 4. Discussion

In this cross-sectional study, we firstly utilized both a posteriori and a priori dietary patterns to investigate the associations between dietary patterns and MetS in a suburban population in Shanghai, China. Our findings demonstrated the inverse associations of the “dairy and fruits” and “coarse cereals and soy products” dietary patterns with MetS. In addition, two a priori dietary pattern scores for DASH and MD were calculated. However, we did not observe any significant correlations between DASH or MD and MetS in the Chinese population.

The prevalence of MetS in our study population was 22.47%, which is similar to the 24.5% reported in a systematic review and meta-analysis of 226,653 Chinese individuals [29], and consistent with the global prevalence of 20–25% [30]. The high prevalence of MetS may be attributed to lifestyle changes in modern society, among which dietary pattern may pose an independent and essential factor in influencing the levels of MetS-related indicators [10,31]. Especially for the Western-style diet high in fat and animal-based foods, a meta-analysis revealed that “Western” dietary patterns were significantly associated with increased MetS risk [32]. Over the past few decades, there has been a significant shift in Chinese dietary patterns and behaviors, with a move from a predominantly plant-based diet to a Western-style diet [33]. Therefore, we generated current dietary patterns in a suburban population in Shanghai and investigated the relationship between these patterns and MetS.

In this study, we observed a protective effect from the “dairy and fruits” dietary pattern on MetS following cluster analysis. High consumption of fruits, egg, and dairy was a crucial component of this pattern. Numerous studies have shown the beneficial effects of fruits and vegetables on chronic diseases, such as MetS and CVD [34,35]. These foods are rich in vitamins, dietary fiber, minerals, and phytochemicals, which contribute to the body’s antioxidant, anti-inflammatory, and electrolyte properties [35]. Similarly, some studies have indicated a negative correlation between egg consumption and the risk of MetS [36,37,38]. Eggs are a source of high-quality protein, unsaturated fatty acids, vitamins, minerals, and bioactive components that can effectively regulate lipid absorption, hepatic lipid metabolism, increase HDL-C levels [39], and improve insulin sensitivity [40]. However, the relationship between dairy consumption and MetS is not consistent. One meta-analysis revealed that a higher intake of dairy products significantly reduced the risk of MetS by 17% in cross-sectional studies and by 14% in cohort studies [41]. Another meta-analysis also reported similar findings for milk and yogurt consumption, but not for cheese consumption [42]. Conversely, Babio et al. suggested that higher cheese consumption was associated with a greater risk of MetS [43]. These inconsistent results may be related to the type of dairy product. Milk and yogurt contain various minerals, such as calcium and potassium, which can reduce fat absorption, resulting in weight and fat loss [44,45], and can also decrease sodium retention, thereby lowering blood pressure [46,47]. Furthermore, whey protein in milk and yoghurt may contribute to reducing endogenous fat, leading to a decline in plasma triglycerides, total cholesterol, and LDL [44]. However, cheese is a high-fat dairy product that loses its whey protein during production, and its phosphorus content, energy density, and sodium are higher than other dairy products. These variations in nutrient content may result in different effects compared to milk or yogurt [43]. Nonetheless, in our study, we were unable to differentiate between the types of dairy products consumed, and this issue requires further investigation.

In our study, we observed a significant negative correlation between the “coarse cereals and soy products” dietary pattern and MetS after factor analysis. Notably, high consumption of coarse cereals is a vital component of this dietary pattern. This finding is consistent with previous research indicating that a low intake of coarse cereals increases the risk of MetS in males, as reported by Cheng et al., based on the China Health and Nutrition Survey [48]. The consumption of coarse cereals has also been shown to promote a healthy gut microbiome, supporting the growth and activity of probiotics [49]. Furthermore, the consumption of coarse cereals has been linked to cardiovascular disease prevention through various pathways, including CaMKII/p-BFAF-3, NF-κB, MAPK, and PI3K/Akt [50]. Another key characteristic of the “coarse cereals and soy products” dietary pattern is the high consumption of soy products and starchy vegetables. Studies have demonstrated that soy products can prevent or improve some symptoms associated with MetS [51,52,53]. Soy products contain essential nutrients, such as protein, polyunsaturated fatty acid, fiber, sterol, and soybean isoflavone, which can reduce glycemic markers (fasting blood glucose and serum insulin levels) [54], improve serum lipids (TG, TC, and LDL-C) [55], blood pressure [56], and flow-mediated dilation [57]. However, the relationship between starchy vegetables, such as potatoes, and MetS is not yet clear. Some studies have suggested that high consumption of potatoes increases the risk of MetS-related diseases, such as type 2 diabetes [58] and hypertension [59], while others have found no significant association between potato or starchy vegetable intake and the risk of obesity, type 2 diabetes, CVD, or MetS [60,61]. This inconsistency may be attributed to differences in cooking methods for starchy vegetables, particularly in Western diets where they are often fried or baked, leading to negative health effects [62,63]. Starchy vegetables are rich in micronutrients and other healthy biological compounds, such as phenolic acid, carotenoid, and resistant starch, which are essential for macronutrient metabolism, antioxidant protection, and chronic disease status [64,65]. Further research is needed to clarify the relationship between starchy vegetable consumption and MetS.

In our study, we evaluated the two a priori dietary patterns, DASH and MD, to explore the association between dietary pattern and MetS. The DASH diet was developed to regulate blood pressure and can also improve various chronic diseases, such as cardiovascular diseases, diabetes, and kidney ailments [66,67,68]. Previous studies have revealed the positive effects of the DASH diet on MetS and its components [69,70,71]. Nonetheless, our findings do not align with these conclusions. Specifically, we found no connection between DASH and MetS, which may be due to differences in ethnic backgrounds across populations. The DASH diet was originally developed in a U.S. population, and the Chinese population is demographically and culturally different from the U.S. population. As reported by Joyce et al. [72], the correlation between DASH and MetS varies across different heritage groups. DASH may be more effective in capturing diet–MetS associations in certain Hispanic/Latino subpopulations, such as central/south Americans. Further investigations are required to identify the underlying reasons. The Mediterranean-style diet is a conventional dietary pattern in countries such as Greece, Italy, and other Mediterranean coastal regions. It emphasizes the use of olive oil as the primary source of fat and moderate wine consumption [73]. Numerous studies have indicated that greater adherence to the Mediterranean diet is associated with a lower risk of MetS in Western populations [74,75,76,77]. However, this association is not as apparent in Asian populations [78]. In our study, we also did not observe a significant correlation between MD and MetS in the Chinese population, which may be due to differences in dietary practices between Western and Eastern countries. Particularly, in our studied population, the consumption of olive oil and grape wine, which are typical components of the Mediterranean diet, was relatively infrequent.

Our study has several limitations that need to be considered. Firstly, in a posterior methods, the final presentation of data does not rely on any prior knowledge, which may introduce subjectivity in the interpretation and naming, and limit the reproducibility and validity of the data. Secondly, seasonal variations may influence the intake of various foods, potentially compromising the reliability of the FFQ data. Furthermore, distinguishing between milk, yogurt, and cheese consumption can be challenging when analyzing the effects of dairy products on MetS using the FFQ. Similar challenges exist for other foods, including fruits. This limitation precludes an in-depth investigation into the impacts of specific types of these foods on MetS. Thirdly, the sample size of this study was relatively small, and study participants were only from a single region, which may not be representative of the wider population. Therefore, future investigations with an expanded sample size and enrolled population from diverse regions are necessary to examine the relationship between dietary patterns and MetS. Finally, the cross-sectional design of the study limits our ability to establish causality between dietary patterns and MetS. Future studies using longitudinal data and intervention trials are expected to better elucidate the causal relationship.

## 5. Conclusion

Our findings suggest that study participants following the “dairy and fruits” and “coarse cereals and soy products” dietary pattern had a protective effect on MetS. Our study recommends increasing the consumption of fruits, coarse cereals, and soy products to reduce the risk of MetS. However, we did not observe any significant correlations between DASH or MD and MetS in the Chinese population. Further exploration is needed to verify the correlation of DASH and MD with MetS in the Chinese population.

## Figures and Tables

**Table 1:** Personal characteristics of study participants between the population with and without MetS.

Variables	With MetS (*n* = 1219)	Without MetS (*n* = 4207)	*p*-Value
Gender ^a^			< 0.001
Male	602(49.38)	1404(33.40)	
Female	617(50.62)	2803(66.63)	
Retirement status ^a^			0.002
Yes	831(68.17)	2667(63.39)	
No	388(31.83)	1540(36.61)	
Marital status ^a^			0.126
Married	1141(93.60)	3883(92.30)	
Other	78(6.40)	324(7.70)	
Education level ^a^			< 0.001
Primary school and below	593(48.65)	1632(38.79)	
Junior high school	419(34.37)	1624(38.60)	
High school and above	207(16.98)	951(22.61)	
Cigarette smoking ^a^			< 0.001
No	829(68.01)	3354(79.72)	
Yes	390(31.99)	853(20.28)	
Alcohol drinking ^a^			< 0.001
No	1014(83.18)	3741(88.92)	
Yes	205(16.82)	466(11.08)	
Tea drinking ^a^			< 0.001
No	710(58.24)	2858(67.93)	
Yes	509(41.76)	1349(32.07)	
Age ^b^	58.31 ± 8.77	55.39 ± 10.22	< 0.001
BMI (kg/m^2^) ^b^	26.93 ± 3.09	23.95 ± 3.00	< 0.001
Energy intake (Kcal/d) ^b^	1378.17 ± 416.41	1393.72 ± 434.84	0.267
Physical activity (MET min/week) ^b^	3458.72 ± 2207.95	3670.39 ± 2157.09	0.003
Waist circumference (cm) ^b^	86.73 ± 8.29	77.28 ± 7.96	< 0.001
Fasting blood glucose (mmol/L) ^b^	6.64 ± 2.06	5.46 ± 1.08	< 0.001
Systolic blood pressure (mmHg) ^b^	146.38 ± 20.85	132.42 ± 21.57	< 0.001
Diastolic blood pressure (mmHg) ^b^	88.34 ± 11.02	81.10 ± 10.87	< 0.001
Triglycerides (mmol/L) ^b^	2.94 ± 1.89	1.50 ± 0.83	< 0.001
HDL cholesterol (mmol/L) ^b^	1.06 ± 0.27	1.43 ± 0.29	< 0.001
LDL cholesterol (mmol/L) ^b^	2.71 ± 0.98	2.80 ± 0.78	0.001

Abbreviations: MetS: metabolic syndrome; SD: standard deviation; MET: metabolic equivalent task. ^a^ Categorical variables were presented as frequency (%); ^b^ continuous variables were reported as mean ± SD.

**Table 2:** Identifying a posteriori dietary pattern based on food intake of study participants by cluster analysis.

Food Groups	Cluster I (*n* = 291)	Cluster II (*n* = 1051)	Cluster III (*n* = 2722)	Cluster IV (*n* = 1362)
Grain	341.84	402.63	311.99	277.44
Coarse cereals	19.01	14.83	10.92	32.72
Starchy vegetables	20.23	19.14	14.41	32.56
Vegetables	220.41	378.58	165.81	235.99
Dark vegetables	129.09	244.55	65.91	115.80
Fruits	137.86	109.17	95.11	168.65
Dairy	125.05	56.24	56.04	149.97
Meats	80.79	81.39	48.75	78.19
Fish	41.88	46.52	27.12	57.66
Other seafood	20.04	16.41	10.14	25.17
Soy products	6.58	5.25	3.18	8.15
Eggs	32.52	37.25	25.45	41.28
Sugared beverages	212.81	8.90	6.20	7.57
Snacks	42.18	27.84	18.94	42.19
Salted food	17.55	18.01	8.56	14.24

Cluster I: snacks and beverages dietary pattern; cluster II: grains and vegetables dietary pattern; cluster III: balanced dietary pattern; cluster IV: dairy and fruits dietary pattern.

**Table 3:** A priori dietary pattern scores of study participants between the population with and without MetS (*n* = 5426).

Dietary Pattern Score, Mean ± SD	Total (*n* = 5426)	With MetS (*n* = 1219)	Without MetS (*n* = 4207)	*p*-Value ^a^
DASH	1.48 ± 1.14	1.46 ± 1.13	1.48 ± 1.14	0.488
MD	3.93 ± 1.59	3.89 ± 1.57	3.95 ± 1.59	0.248

Abbreviations: DASH: dietary approaches to stop hypertension; MD: Mediterranean diet. ^a^ *p*-value for *t*-test.

**Table 4:** A posteriori dietary patterns in association with the risk of MetS using non-conditional logistic regression models.

Method	Dietary Pattern	Model 1		Model 2	
		OR (95 % CI)	*p*-Value	OR (95 % CI)	*p*-Value
Cluster analysis	Balanced pattern	Reference		Reference	
	Snacks and beverages pattern	0.92 (0.69, 1.23)	0.580	1.06 (0.77, 1.46)	0.718
	Grains and vegetables pattern	1.03 (0.88, 1.22)	0.699	0.97 (0.81, 1.17)	0.764
	Dairy and fruits pattern	0.66 (0.56, 0.78)	<0.001	0.81 (0.66, 0.98)	0.032
Factor analysis ^a^	High protein pattern				
	Q1	Reference		Reference	
	Q2	0.99 (0.82, 1.18)	0.864	1.02 (0.85, 1.22)	0.838
	Q3	0.85 (0.70, 1.01)	0.070	0.91 (0.75, 1.10)	0.318
	Q4	0.99 (0.83, 1.19)	0.936	1.12 (0.93, 1.36)	0.239
	*p*-value for trend ^b^	0.660		0.382	
	Grains and vegetables pattern				
	Q1	Reference		Reference	
	Q2	0.94 (0.78, 1.12)	0.467	0.95 (0.79, 1.15)	0.616
	Q3	1.05 (0.88, 1.26)	0.579	1.07 (0.89, 1.29)	0.453
	Q4	0.93 (0.78, 1.12)	0.433	0.92 (0.76, 1.12)	0.406
	*p*-value for trend ^b^	0.622		0.553	
	Coarse cereals and soy products pattern				
	Q1	Reference		Reference	
	Q2	0.81 (0.68, 0.97)	0.022	0.86 (0.72, 1.03)	0.098
	Q3	0.79 (0.66, 0.94)	0.009	0.86 (0.71, 1.03)	0.105
	Q4	0.67 (0.56, 0.80)	<0.001	0.74 (0.61, 0.91)	0.005
	*p*-value for trend ^b^	<0.001		0.007	
	Snacks and beverages pattern				
	Q1	Reference		Reference	
	Q2	1.09 (0.91, 1.31)	0.333	1.08 (0.90, 1.30)	0.417
	Q3	0.99 (0.82, 1.18)	0.890	1.02 (0.85, 1.24)	0.822
	Q4	0.99 (0.82, 1.19)	0.898	1.09 (0.88, 1.34)	0.422
	*p*-value for trend ^b^	0.593		0.537	
	Dairy and fruits pattern				
	Q1	Reference		Reference	
	Q2	0.82 (0.69, 0.98)	0.025	0.97 (0.80, 1.16)	0.707
	Q3	0.69 (0.57, 0.82)	<0.001	0.95 (0.78, 1.15)	0.598
	Q4	0.60 (0.50, 0.72)	<0.001	0.97 (0.79, 1.19)	0.753
	*p*-value for trend ^b^	<0.001		0.722	

Note: ^a^ factor scores were divided into Q1, Q2, Q3, and Q4 according to the quartiles; ^b^ test for trend based on variable containing median value for each quartile. Model 1: unadjusted model; model 2: adjusted for age, energy intake, physical activity, gender, education level, retirement status, smoking behavior, alcohol drinking, and tea drinking.

**Table 5:** A priori dietary patterns in association with the risk of MetS using non-conditional logistic regression models.

Dietary Pattern Score	Model 1		Model 2	
	OR (95 % CI)	*p*-Value	OR (95 % CI)	*p*-Value
DASH	0.98 (0.93, 1.04)	0.488	1.02 (0.96, 1.08)	0.541
MD	0.98 (0.94, 1.02)	0.247	1.01 (0.97, 1.06)	0.675

Abbreviations: DASH: dietary approaches to stop hypertension; MD: Mediterranean diet. Model 1: unadjusted model; model 2: adjusted for age, energy intake, physical activity, gender, education level, retirement status, smoking behavior, alcohol drinking, and tea drinking.

## Data Availability

The data presented in this study are available on request from the corresponding author. The data are not publicly available due to privacy of the study participants.

## Supplementary Materials

The following supporting information can be downloaded at: https://www.mdpi.com/article/10.3390/nu15092185/s1, Table S1: DASH diet scoring system; Table S2: MD scoring system; Table S3: food groupings used in dietary pattern analysis; Table S4: factor loadings and dietary patterns for the 15 food groups derived from factor analysis.
