# Supplementary material for: The Effect of Dietary Pattern on Metabolic Syndrome in a Suburban Population in Shanghai, China
Source: Nutrients. 2023 May 4;15(9):2185. doi: 10.3390/nu15092185 (PMC10181156; doi:10.3390/nu15092185)
Supplement: Supplementary file 1 [file nutrients-15-02185-s001.zip › nutrients-2362212-supplementary.pdf]

Supplementary Table S1. DASH diet scoring system.

Nutrient	DASH diet nutrient composition [a]	DASH score target (1)	Intermediate target (2)
Saturated fat	6% of energy	6% of energy	11% of energy
Total fat	27% of energy	27% of energy	32% of energy
Protein	18% of energy	18% of energy	16.5% of energy
Cholesterol	150 mg	71.4 mg/1000 kcal	107.1 mg/1000 kcal
Fiber	31 g	14.8 g/1000 kcal	9.5 g/1000 kcal
Magnesium	500 mg	238 mg/1000 kcal	158 mg/1000 kcal
Calcium	1240 mg	590 mg/1000 kcal	402 mg/1000 kcal
Potassium	4700 mg	2238 mg/1000 kcal	1534 mg/1000 kcal
Sodium [b]	2400 mg	1143 mg/1000 kcal	1286 mg/1000 kcal

Abbreviation: DASH, Dietary Approaches to Stop Hypertension trial. [a] based on a 2100-kcal diet; [b] sodium target based on the Sixth Report of the Joint National Committee on Prevention, Detection, Evaluation, and Treatment of High Blood Pressure recommendations.

Supplementary Table S2. MD scoring system.

Component	Intake of minimum score	Intake of maximum score	Score range
Vegetables (g)	Below the median	Above the median	0-1
Fruits (g)	Below the median	Above the median	0-1
Nuts (g)	Below the median	Above the median	0-1
Whole grains (g)	Below the median	Above the median	0-1
Legumes (g)	Below the median	Above the median	0-1
Fish and seafood (g)	Below the median	Above the median	0-1
Monounsaturated/saturated fat ratio	Below the median	Above the median	0-1
Red and processed	Above the	Below the median	0-1

meats (g)	median		
Alcohol (g)	Females		
	<5, >15	5-15	0-1
	Males		
	<15, >25	15-25	0-1

Abbreviation: MD, Mediterranean Diet.

Supplementary Table S3. Food groupings used in dietary pattern analysis.

Food Groups	Components
Grain	Rice, bread, cake, noodle, flour, pizza
Coarse cereals	Millet, sorghnm, oats
Starchy vegetables	Potato, cassava, yam
Vegetables	Leafy greens, tomato, green beans, mushroom
Dark vegetables	Carrot, pepper, tomato, spinach
Fruits	Fresh and canned (no added sugar) fruits
Dairy	Whole milk, skim milk, flavored milk, cheese, yogurt
Meats	Chicken, duck meat, pork, beef, lamb, animal viscera
Fish	Freshwater fish, sea fish
Other seafood	Shrimp, crab, shellfish
Soy products	Soybean, soy milk, tofu
Eggs	Whole eggs, yolk, white, preserved eggs
Sugared beverages	Carbonated drinks, fruit or flavored drinks, soft drinks
Snacks	Cakes, chocolate, mooncake, potato chips, ice cream
Salted food	Preserved meat, preserved vegetables, sausages

Supplementary Table S4. Factor loadings and dietary patterns for the 15 food groups derived from factor analysis.

Food Groups	Factor I	Factor II	Factor III	Factor IV	Factor V
Grain	-	0.21	-	-	-0.68
Coarse cereals	-	-	0.69	-	-

Starchy vegetables	-	-	0.71	-	-
Vegetables	-	0.81	-	-	-
Dark vegetables	-	0.83	-	-	-
Fruits	-	-	0.41	-	0.37
Dairy	-	-	-	-	0.66
Meats	0.48	-	-	0.35	-0.29
Fish	0.77	-	-	-	-
Other seafood	0.75	-	-	-	-
Soy products	0.30	-	0.39	0.22	-
Eggs	0.27	-	-	-	-
Sugared beverages	-	-	-	0.67	-
Snacks	-	-	-	0.56	0.21
Salted food	-	-	-	0.57	-

Factor loadings of < 0.2 in absolute terms were excluded for simplicity; factor I: high protein dietary pattern; factor II: grains and vegetables dietary pattern; factor III: coarse cereals and soy products dietary pattern; factor IV: snacks and beverages dietary pattern; factor V: dairy and fruits dietary pattern.
